# Sacubitril/valsartan versus valsartan in regressing myocardial fibrosis in hypertension: a prospective, randomized, open-label, blinded endpoint clinical trial protocol

**DOI:** 10.3389/fcvm.2023.1248468

**Published:** 2023-08-22

**Authors:** Vivian Lee, Qishi Zheng, Desiree-Faye Toh, Chee Jian Pua, Jennifer A. Bryant, Chi-Hang Lee, Stuart A. Cook, Javed Butler, Javier Díez, A. Mark Richards, Thu-Thao Le, Calvin W. L. Chin

**Affiliations:** ^1^ National Heart Research Institute Singapore (NHRIS), National Heart Centre Singapore, Singapore, Singapore; ^2^ Cochrane Singapore, Singapore, Singapore; ^3^ Department of Cardiology, National Heart Centre Singapore, Singapore, Singapore; ^4^ Department of Cardiology, National University Heart Centre Singapore, Singapore, Singapore; ^5^ Cardiovascular & Metabolic Disorders Program, Duke-NUS Medical School, Singapore, Singapore; ^6^ Baylor Scott and White Research Institute, Dallas, TX, United States; ^7^ Department of Medicine, University of Mississippi School of Medicine, Jackson, MS, United States; ^8^ Centre for Applied Medical Research (CIMA), and School of Medicine, University of Navarra, Pamplona, Spain; ^9^ Center for Network Biomedical Research of Cardiovascular Diseases (CIBERCV), Carlos III Institute of Health, Madrid, Spain; ^10^ Cardiovascular Research Institute, National University Heart Centre, Singapore, Singapore; ^11^ Christchurch Heart Institute, University of Otago, Christchurch, New Zealand; ^12^ Cardiovascular Academic Clinical Program (ACP), Duke-NUS Medical School, Singapore, Singapore

**Keywords:** myocardial fibrosis, sacubitril/valsartan, ARNi, hypertensive heart disease, heart failure, cardiovascular magnetic resonance imaging, biomarkers

## Abstract

**Background:**

Diffuse interstitial myocardial fibrosis is a key common pathological manifestation in hypertensive heart disease (HHD) progressing to heart failure (HF). Angiotensin receptor–neprilysin inhibitors (ARNi), now a front-line treatment for HF, confer benefits independent of blood pressure, signifying a multifactorial mode of action beyond hemodynamic regulation. We aim to test the hypothesis that compared with angiotensin II receptor blockade (ARB) alone, ARNi is more effective in regressing diffuse interstitial myocardial fibrosis in HHD.

**Methods:**

Role of ARNi in Ventricular Remodeling in Hypertensive LVH (REVERSE-LVH) is a prospective, randomized, open-label, blinded endpoint (PROBE) clinical trial. Adults with hypertension and left ventricular hypertrophy (LVH) according to Asian sex- and age-specific thresholds on cardiovascular magnetic resonance (CMR) imaging are randomized to treatment with either sacubitril/valsartan (an ARNi) or valsartan (an ARB) in 1:1 ratio for a duration of 52 weeks, at the end of which a repeat CMR is performed to assess differential changes from baseline between the two groups. The primary endpoint is the change in CMR-derived diffuse interstitial fibrosis volume. Secondary endpoints include changes in CMR-derived left ventricular mass, volumes, and functional parameters. Serum samples are collected and stored to assess the effects of ARNi, compared with ARB, on circulating biomarkers of cardiac remodeling. The endpoints will be analyzed with reference to the corresponding baseline parameters to evaluate the therapeutic effect of sacubitril/valsartan vs. valsartan.

**Discussion:**

REVERSE-LVH will examine the anti-fibrotic potential of sacubitril/valsartan and will offer mechanistic insights into the clinical benefits of sacubitril/valsartan in hypertension in relation to cardiac remodeling. Advancing the knowledge of the pathophysiology of HHD will consolidate effective risk stratification and personalized treatment through a multimodal manner integrating complementary CMR and biomarkers into the conventional care approach.

**Clinical Trial Registration**: ClinicalTrials.gov, identifier, NCT03553810.

## 1. Introduction

Hypertensive heart disease (HHD) is manifested in a range of cardiac morphological and functional derangements including the development of left ventricular hypertrophy (LVH). Beyond structural compensation (increased LV wall thickening and LV mass) due to hemodynamic effects of sustained elevated blood pressure, a complex interplay between co-morbidities and neurohormonal status appears to contribute to the maladaptive progression, leading to cardiomyocyte hypertrophy, cell death, alterations in the coronary microcirculation, and diffuse interstitial fibrosis (1–3). These changes eventually lead to LV dysfunction and potentially to overt heart failure (HF). Thus, pharmacological therapies fostering regression of myocardial fibrosis play a vital role as a beneficial intervention for HHD.

Conventional anti-hypertensive therapies which suppress the renin–angiotensin–aldosterone system (RAAS) including angiotensin-converting enzyme inhibitors (ACEi) or angiotensin receptor blockers (ARB) have been shown to regress myocardial fibrosis on biopsy (4, 5).

Angiotensin receptor–neprilysin inhibitor (ARNi) is a new class of drug that blocks the RAAS and augments natriuretic peptides. It is a dual agent comprising a neprilysin inhibitor (sacubitril) and an ARB (valsartan). Sacubitril/valsartan has shown superior benefits over conventional ARB or ACEi monotherapy in terms of blood pressure lowering (6), clinical outcome improvement (7), and reduction in cardiac wall stress and injury biomarkers (8–10) and is endorsed in international guidelines for the treatment of HF (11, 12). Pre-clinical data has suggested that sacubitril/valsartan has superior efficacy in promoting the regression of myocardial fibrosis compared with valsartan alone in HF with diabetes (13).

In patients with hypertension, a 52-week treatment with sacubitril/valsartan has demonstrated a greater reduction in LV mass, measured by cardiovascular magnetic resonance (CMR), compared with the ARB olmesartan (14). Importantly, this reduction in LV mass appears to be independent of changes in blood pressure. Similarly, ventricular remodeling regression irrespective of blood pressure control was observed in perimenopausal women treated with sacubitril/valsartan for 24 weeks who showed greater reduction in indexed LV mass on echocardiography, compared with those on valsartan. The accompanying reduction of fibrosis-related serum biomarkers (TGF-β, CT-GF, and α-SMA) suggested the possible involvement of fibrosis in cardiac remodeling (15). Therefore, direct examination of therapy-effected regression of fibrosis on CMR imaging, along with existing evidence, would consolidate the role of fibrosis in myocardial remodeling and its modulation by ARNi.

In REVERSE-LVH, we aim to compare the changes in diffuse interstitial myocardial fibrosis with respect to LVH regression in HHD patients undergoing sacubitril/valsartan vs. valsartan treatment alone. We hypothesize that 52 weeks of sacubitril/valsartan therapy will result in greater regression of diffuse interstitial myocardial fibrosis and LV mass relative to valsartan therapy, independent of blood pressure control. With the advances of CMR, diffuse interstitial myocardial fibrosis can now be measured non-invasively using T1 mapping and extracellular volume (ECV) quantification (16).

## 2. Methods

### 2.1. Study design and population

REVERSE-LVH is a prospective, randomized, open-label, blinded endpoint (PROBE) clinical trial, designed to compare the effects of 52 weeks of treatment with sacubitril/valsartan (an ARNi class drug) with valsartan (an ARB class drug) on the primary endpoint of change in interstitial volume measured by CMR in patients with hypertension and LVH. The secondary endpoints include changes in LV mass, volumes, and function measured by CMR from baseline.

Patients with hypertension and LVH diagnosed according to age- and sex-specific CMR thresholds in Asians (17) are eligible. Participants who were previously recruited to the REMODEL study (our ongoing prospective study that examines myocardial fibrosis in hypertension; ClinicalTrials.gov Identifier: NCT02670031) are screened for eligibility. This cohort consists of participants from community clinics and two major tertiary heart centers in Singapore: National Heart Centre Singapore (NHCS) and National University Heart Centre, Singapore (NUHCS). No restrictions based on ethnicity or gender are imposed. Details of the inclusion and exclusion criteria are listed in Table 1. Written informed consent is obtained from all participants. Informed consent, randomization, all study visits, and procedures are carried out at NHCS.

**Table 1:** Inclusion and exclusion criteria.

Inclusion criteria
1. Age ≥21 years of age
2. Physician diagnosed essential hypertension, on at least one medication for blood pressure control (including newly diagnosed hypertension or resistant hypertension)
3. Increased LV mass on CMR (based on local age- and sex-specific CMR ranges)
Exclusion criteria
1. Known secondary causes of hypertension
2. Other causes of LVH (such as hypertrophic and infiltrative cardiomyopathy)
3. Previous intolerance to ARB
4. History of heart failure
5. History of cardiovascular events (myocardial infarction, strokes, and transient ischemic attacks)
6. Known atrial fibrillation
7. History or presence of any other disease with a life expectancy of <3 years
8. Stage IV/V chronic renal disease (eGFR of <30 mL/min/1.73 m^2^)
9. Serum potassium of >5.2 mmol/L (mEq/L) at visit 1
10. Unable to commit to study follow-up
11. Unable to understand or comply with study procedures (including CMR)
12. Pregnant or nursing (lactating) women

ARB, angiotensin II receptor blocker; CMR, cardiovascular magnetic resonance; eGFR, estimated glomerular filtration rate; LV, left ventricular; LVH, left ventricular hypertrophy.

### 2.2. Randomization, blinding, and treatment

**Figure 1:**
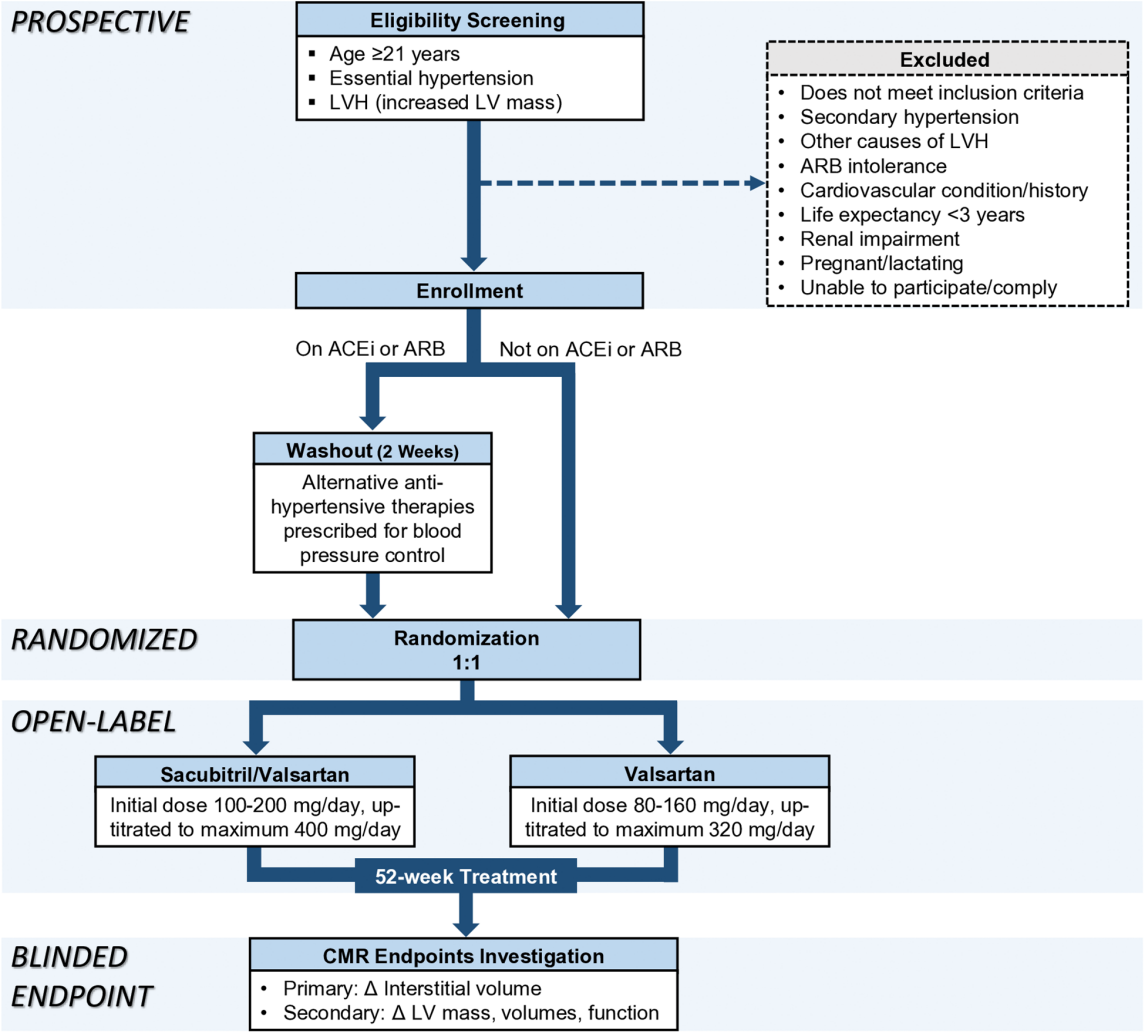
Recruitment, randomization, and treatment protocol. ACEi, angiotensin-converting enzyme inhibitor; ARB, angiotensin II receptor blocker; LV, left ventricular; LVH, left ventricular hypertrophy; Δ, change.

To eliminate possible confounding or residual effects from RAAS suppression by anti-hypertensive medications that some participants might be taking at the time of enrollment, participants who are on ACEi or ARB will undergo a 2-week washout period prior to commencing their respective assigned therapies. This duration of washout period is consistent with ARNi studies (6, 18) and is more than five elimination half-lives of commonly used ACEi and ARB (19, 20). Alternative therapies or up-titration of other anti-hypertensive agents (including but not limited to amlodipine and/or hydrochlorothiazide) are prescribed to control blood pressure during the 2-week washout period (Figure 1).

Participants are randomized on an allocation ratio of 1:1 to either sacubitril/valsartan or valsartan without blinding. Randomization service is provided by the Singapore Clinical Research Institute (SCRI) in closed, opaque, and sequentially numbered envelopes. Research member who measures CMR endpoints is blinded to treatment allocation and clinical details including blood pressure values.

The treatment duration is 52 weeks, with the same target systolic blood pressure for both treatment groups which is less than 140 mmHg according to contemporary guidelines at the time of study conception (21, 22). The initial dosage of sacubitril/valsartan is 100 mg–200 mg/day and can be titrated up to a maximum dose of 400 mg/day. The initial dosage of valsartan is 80 mg–160 mg/day and can be titrated to a maximum dose of 320 mg/day. Medications are titrated to the maximum dose tolerated by the participants. If the study medications are inadequate for optimal blood pressure control, additional non-RAAS inhibiting anti-hypertensive agents are prescribed. Study medications are dispensed by a pharmacist at NHCS. Treatment compliance is monitored by pill counting at each clinic visit.

Participants may withdraw from the study at any time. Withdrawal can be initiated and documented by the investigator if continuation would be detrimental to the participant’s well-being. If withdrawal happens after more than 12 weeks of randomized treatment, all endpoint investigations will be performed at the time of withdrawal (unless participant is not contactable, refused, or deceased). The first participant was recruited on 19 June 2019.

### 2.3. Study procedures

The study is organized into five scheduled clinic visits and two phone calls as outlined in Table 2. Additional visits are arranged if necessary. At baseline visit, demographics (age, sex, race), past medical history, and anthropometric measurements (height, weight) are collected. The EuroQol EQ-5D-3L questionnaire is used to evaluate five aspects of health-related quality of life (mobility, self-care, usual activities, pain/discomfort, and anxiety/depression) at baseline, visit 4 and visit 5.

**Table 2:** Study visits and procedures.

Visit	Washout	1	2	3		4		5
Week	−2	0	4^a^	8^a^	12^a^	26^a^	38^a^	52
Informed consent taking	×^b^	×^b^			Phone call follow-up		Phone call follow-up	
Demographics and anthropometrics collection	×^b^	×^b^						
Collection of past medical history, including medications	×^b^	×^b^						
Urine pregnancy test	×	×	×	×		×		×
Randomization		×						
Health-related quality of life assessment		×				×		×
Study medication dispense		×	×	×		×		
Medication adherence check		×	×	×		×		×
Blood collection for renal function and potassium level		×	×	×		×		×
Blood collection for hematocrit		×						×
Blood collection for storage		×						×
Office blood pressure measurement		×	×	×		×		×
24-h ambulatory blood pressure monitoring		×	×	×		×		×
Cardiovascular magnetic resonance		×						×
Adverse events evaluation		×	×	×		×		×

^a^ Additional clinic visits are arranged if necessary.

^b^ These procedures are only done once, either at the washout visit or visit 1.

#### 2.3.1. Urine pregnancy test

Women who are not pregnant at enrollment but are otherwise of child-bearing potential will undergo urine dip-stick pregnancy test at each study visit. A positive urine pregnancy test would require study drug discontinuation and participant’s withdrawal from the study.

#### 2.3.2. Blood pressure measurement

A correctly sized cuff of the OnTrak 90277 device (Spacelabs Healthcare, Snoqualmie, WA, USA) is applied to the participant’s non-dominant arm. Over 24 h, automated out-of-clinic blood pressure measurements are taken at intervals of 20 min from 6 AM to 10 PM and 30 min from 10 PM to 6 AM the following day. Instruction and demonstration on the device usage are given to the participants by the clinical research coordinator.

#### 2.3.3. Blood collection and storage

Serum creatinine and potassium levels are measured at all visits as part of safety monitoring. Additional serum and plasma are collected and stored in aliquots at −80°C for future evaluation of circulating biomarkers pertaining to RAAS activity, myocardial fibrosis (including pro-fibrotic mediators and parameters assessing cardiac extracellular matrix turnover), and injury. This will be explored and reported in a separate circulating biomarkers sub-study.

#### 2.3.4. Cardiovascular magnetic resonance imaging

##### 2.3.4.1. Image acquisition

All CMR scans are performed using a standardized imaging protocol on the Siemens Aera 1.5 T scanner (Siemens Healthineers, Erlangen, Germany). Long-axis balanced steady-state free precession cine images are acquired in the two-, three-, and four-chamber views (acquired voxel size 1.6 × 1.3 × 8.0 mm; 30 phases per cardiac cycle). Short-axis cines extending from the mitral valve annulus to the apex are also acquired (acquired voxel size 1.6 × 1.3 × 8.0 mm; 30 phases per cardiac cycle).

Diffuse interstitial myocardial fibrosis is assessed by myocardial T1 mapping using the modified Look-Locker inversion-recovery sequence. Native T1 map is acquired using a heartbeat scheme of 5(3)3; post-contrast T1 map is acquired 15 min after administration of 0.1 mmol/kg of gadobutrol (Gadovist; Bayer Pharma AG, Germany) using a heartbeat scheme of 4(1)3(1)2.

##### 2.3.4.2. Image analysis

All images are de-identified and analyzed at the National Heart Research Institute (NHRIS) CMR Core Lab by trained personnel blinded to trial data, including treatment allocation and blood pressure values.

Analysis of LV mass, volumes, and function (including multi-directional myocardial strain) is performed using standardized protocols (17, 23). ECV fraction is calculated using the T1 mapping module in cvi42 image analysis software (Circle Cardiovascular Imaging, Calgary, Canada) (24). Interstitial volume (indexed to body surface area) is derived from ECV × myocardial volume, where myocardial volume (mL) is defined as myocardial mass(g)/1.05 g/mL. Hematocrit for calculating ECV is taken on the day of CMR.

#### 2.3.5. Adverse events evaluation

At each clinic visit, adverse events related to study treatment and/or procedures are assessed. If present, they will be recorded and reported to the local ethics board and Health Sciences Authority (the local governing body which oversees clinical trials) within the stipulated timeframe.

### 2.4. Statistical consideration

#### 2.4.1. Sample size calculation

We determined that a sample size of 35 participants per group would provide the trial with 80% power at a 5% significance level (two-sided) to detect an absolute minimum difference between treatment groups of 3.5 mL/m^2^ in terms of change in interstitial volume from baseline following 52 weeks, assuming an SD of 5.8 mL/m^2^ [data that was subsequently published (25)] and a moderate correlation of 0.60 between interstitial volumes at baseline and 1 year. The effect size was based on an estimate of the magnitude of myocardial fibrosis regression that could be expected to translate into improved clinical outcomes (5). The study plan is to randomize 80 participants, allowing up to 15% dropout and treatment discontinuation.

#### 2.4.2. Statistical analysis

The analyses will be based on the intention-to-treat principle, such that all participants will be included in analysis according to their assigned treatment groups.

Baseline characteristics and changes in CMR endpoints between the two treatment groups will be compared. If significant baseline differences are found between the two groups, appropriate modifications to analytical methods may be made to adjust for the differences. Categorical variables will be compared with *χ*^2^ test and described as count (percentage). Continuous variables that are normally distributed will be compared using the parametric Student’s *t*-test and presented as mean ± SD. If the normality assumption is not met, they will be compared using the non-parametric Mann–Whitney *U* test and described as median (interquartile range). All analyses will be performed two-sided at the 5% significance level. A *p*-value of <0.05 will be considered statistically significant. Additional exploratory analyses may be performed.

Statistical analyses will be performed using SPSS (IBM SPSS Inc., NY, USA) and GraphPad Prism (GraphPad Software, Inc., CA, USA).

## 3. Discussion

### 3.1. Significance

To the best of our knowledge, REVERSE-LVH is the first trial that examines the anti-fibrotic potential of sacubitril/valsartan in a clinical population, guided by imaging. Whilst pre-clinical data support that sacubitril/valsartan combination is more effective in regressing hypertrophy and fibrosis compared with ARB such as valsartan (13, 26), our work will verify such benefits in the clinical setting. Importantly, this study will provide possible mechanistic insights into the clinical benefits of sacubitril/valsartan in HF as observed in the pivotal PARADIGM-HF (7) and PARAGON-HF (27) trials.

Myocardial fibrosis, a hallmark of HF, has shown regression on histology with lisinopril and losartan in HHD (4, 5), torasemide in hypertensive HF (28), and spironolactone in non-hypertensive HF (29). Building upon these findings, REVERSE-LVH aims to further enhance our understanding of HHD by focusing on treatments specifically targeting myocardial fibrosis. As this field advances, new therapeutic targets will emerge, and more specific agents will be developed. The role of CMR in monitoring disease progression and evaluating treatment response is expected to evolve, providing valuable insights into the effectiveness of fibrosis-targeted therapies. Our subsequent exploration of circulating fibrosis biomarkers would potentially complement the imaging evidence with regard to mechanism elucidation as well as improvement of treatment monitoring strategy via a multi-marker approach.

Our study population comprises hypertensive individuals with LVH free from overt HF and other cardiovascular conditions. We have demonstrated in the REMODEL study that 28% of adults with hypertension have LVH. The proportion of LVH doubles in the presence of both hypertension and diffuse interstitial fibrosis (24). This, along with the notion that LVH confers increased risk of HF, highlights the substantial hypertensive population that will potentially benefit from the findings of the REVERSE-LVH trial.

### 3.2. Design

This trial is conducted in a PROBE design that is more reflective of real-world clinical practice, less costly whilst delivering the advantages of classic double-blinded studies (30). Although a double-blinded design is maximally robust with respect to bias, it requires the commercial production of blinded medications and also adds difficulty to titration of medications as treating clinicians are blinded from the medication allocation and dosages. A PROBE study design, used in many hypertension trials, involves treatment known to participants and clinicians as in standard care practice, enables scientific investigation with stringent randomization, and eliminates bias with blinded endpoint evaluation.
